# Colorimetric Indicators Based on Anthocyanin Polymer Composites: A Review

**DOI:** 10.3390/polym14194129

**Published:** 2022-10-02

**Authors:** Despoina Kossyvaki, Marco Contardi, Athanassia Athanassiou, Despina Fragouli

**Affiliations:** 1 Smart Materials, Istituto Italiano di Tecnologia, Via Morego 30, 16163 Genova, Italy; 2 Dipartimento di Informatica Bioingegneria, Robotica e Ingegneria dei Sistemi (DIBRIS), Università degli Studi di Genova, Via Opera Pia 13, 16145 Genova, Italy

**Keywords:** pH indicators, food packaging, antioxidant, wound management, nanofibers, natural pigments

## Abstract

This review explores the colorimetric indicators based on anthocyanin polymer composites fabricated in the last decade, in order to provide a comprehensive overview of their morphological and compositional characteristics and their efficacy in their various application fields. Notably, the structural properties of the developed materials and the effect on their performance will be thoroughly and critically discussed in order to highlight their important role. Finally, yet importantly, the current challenges and the future perspectives of the use of anthocyanins as components of colorimetric indicator platforms will be highlighted, in order to stimulate the exploration of new anthocyanin sources and the in-depth investigation of all the possibilities that they can offer. This can pave the way for the development of high-end materials and the expansion of their use to new application fields.

## 1. Introduction

Nowadays, people’s fast-paced lifestyle imposes efficient and straightforward quality testing of products and physiological processes in order to readily assure the adequate protection from toxins, bacteria and microbes. With the continuous growth of the global population, the increasing environmental pollution and the constant reduction of the natural resources, the safe and constant monitoring of food and water quality, as well as of health conditions has become extremely significant. Among the quality evaluation processes, the immediate acquisition of information of the microbial load within a specific macro and microenvironment is of emerging importance. It may prevent the consumption of spoiled food and polluted water and may also reveal various skin infections or administration of wrong medication.

Therefore, there is the need for microbial growth monitoring through straightforward and directly applicable tools that can be used also by the end-users, for the immediate identification of the aliment, water or health condition state, without the requirement of any pre-treatment process and without the need of complicated instrumentation. Since the metabolic products of microorganisms can alter the pH of the environment in which they live and multiply [1], the fabrication of high performant pH indicators is possible, through visual colorimetric changes, able to indicate the microbes’ presence with qualitative or semi-quantitative information [2].

In the last years, various colorimetric indicators have been developed, based on the incorporation of substances that change their color upon pH changes, within polymers [3]. The polymer-based indicators are gaining ground since they can be developed into multiple, stable and easily handled forms (e.g., films, fiber-mats, etc.); they have mechanical stability and ductility; and they are lightweight. Above all, the polymer-based indicators preserve or even improve the performance of the englobed pH-sensitive molecules, while, in some cases, the polymer matrix enhances their stability as it protects them from oxidation or other environmental factors [4,5,6,7,8]. Some polymer-based indicators are already patented and commercialized (e.g., FreshTag^®^, SensorQ^®^, Food Sentinel System^®^, Toxin Guard^®^ and RipeSense^®^ [9,10,11,12] (http://www.ripesense.co.nz/ (accessed on 1 June 2022)) mainly for smart food packaging applications. 

In most of the colorimetric polymer-based indicators developed in the previous years, synthetic pH-sensitive reagents such as methyl red and bromothymol blue [13], congo red [14], bromophenol blue [15], chlorophenol red [16], bromothymol blue, bromocresol green and phenol red [17], as well as dyes specifically synthesized for this aim, such as the GJM-534 [18], have been used. However, their use is questionable for applications in food and health due to the possibility of causing several detrimental effects to humans, including carcinogenesis and mutagenesis [19,20,21]. In addition, in case such materials end up in the environment, they may pollute the soil, groundwater, and the oceans, causing several toxic effects to diverse living organisms [22]. As a result, scientists recently focused on the use of natural and biocompatible pH sensitive dyes, such as curcumin [23], alizarin [24,25,26], litmus [27], naphthoquinone [28,29], *Spirulina* sp. [30], or anthocyanins [31,32,33], with the latter to be the most popular for such applications. 

**Figure 1:**
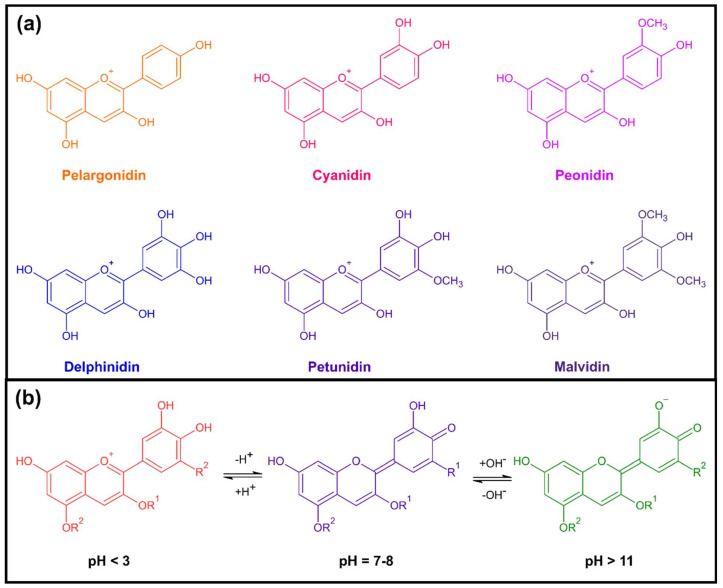
(**a**) The 6 main anthocyanidins and (**b**) the structure of pH changing anthocyanins. The color with which the structures are represented in the figure is resembling their real one.

Anthocyanins are water and alcohol soluble non-toxic flavonoids which are abundant in plants. Structurally, they are substituted glycosides and acylglycosides of 2-phenylbenzopyrilium salts (anthocyanidins). Their name derives from the Greek words *άνθος* (ànthos) that means flower, and *κυανός* (kianòs) that means blue. Depending on the source, environment, cultivation techniques and maturity stage, the anthocyanin content ranges between 0.1% and 1.0% of the dry weight of the plant [34]. Anthocyanidins’ basic structure is composed of the C_6_—C_3_—C_6_ structural backbone called flavylium cation [35] and, depending on the number and position of the hydroxyl and/or methyl ether groups attached, they are of different types. Although more than 30 anthocyanidins are identified up to date, 90% of the naturally occurring anthocyanins are based on only six structures (30% is based on cyanidin 2, 22% on delphinidin 3, 18% on pelargonidin 1 and the remaining 20% on peonidin 4, malvidin 6 and petunidin 5) (Figure 1a) [36,37]. Depending on the hydroxylation and methylation of the flavonoid skeleton, the color hue of these components range from orange-red (pelargonidin) to blue-violet (delphinidin).

The coloration of anthocyanins alters reversibly when exposed to an environment with a different pH [38,39]. Specifically, upon protonation/deprotonation, their delocalized electronic structure rearranges and the change of the total number of resonant electrons as well as their confinement result in changes of their color [40]. As shown in Figure 1b, in a highly acidic pH (pH 1–3) the predominant tautomeric form is the flavylium cation, giving the pigment a red coloration. An increase in the pH results in kinetic and thermodynamic competition between the hydration of the flavylium cations and the proton transfer reactions of their acidic hydroxyl groups. Upon hydration of the flavylium cations, colorless carbinol pseudo bases are formed, which may undergo ring opening and the formation of yellow retrochalcones. Proton transfer reactions give rise to more violet quinonoidal bases. At pH values between 4 and 6, a mixture of equilibrium forms of anthocyanins, such as the red colored flavylium cations, purple or blue colored quinonoidal bases, colorless carbinol pseudo bases, and yellow colored chalcones are formed, leading to an overall violet color. At pH values between 6 and 8, there is a further deprotonation of the quinonoidal bases resulting in the formation of more bluish resonance-stabilized quinonoid anions (anionic quinoidal base). Finally, at pH ≥ 9, the color turns progressively to green and yellow tones due to the further deprotonation of the molecule and the presence of the dianionic quinoidal base (green/yellowish green), chalcone, chalcone base (yellow) and carbinol pseudo base [31].

**Figure 2:**
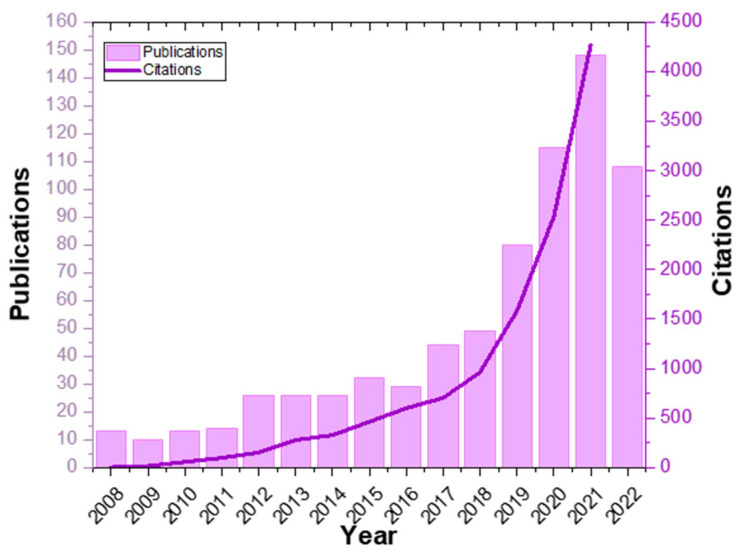
The number of publications and citations for indicators with anthocyanins, as it has evolved during the last years (2008–2022). (Search query in Web of Science: (ALL = (anthocyanins)) AND ALL = (indicators), retrieved 1 September 2022).

Due to this property, anthocyanins are considered an extremely promising group of compounds for use in polymeric pH colorimetric indicators, and in the last years, considerable attention has been paid to the development and study of such systems (Figure 2). Apart from the studies on specific anthocyanin-based materials, a notable number of reviews has been also published, mainly focused on the anthocyanins’ chemistry [41,42], biological properties and their use in nutrition and pharmaceutics [43,44,45], extraction techniques [46], their (micro/nano) encapsulation processes [35,47,48], their role in active packaging [49] but also their color changes [50]. Although the reviews focus on the abovementioned properties of anthocyanins and their use in pH colorimetric films mainly for food packaging applications [51,52,53,54], to the authors’ knowledge, not enough attention has been paid to the different structures of the polymer-anthocyanins composite systems and what role they can play in the pH responsivity, colorimetric properties and effectiveness of the developed systems. The latter needs to be thoroughly and critically discussed since the scope of the fabrication of these systems is not only the demonstration of their color change, but mainly the conception of the color changes also by inexperienced users and the real-time monitoring of the targeted environment.

The current review sets its focus on anthocyanin-based polymer composite materials used as pH-indicators. First, the main application fields are explored, underlining the importance of indicators for modern societies. The anthocyanin sources and the ways of the molecules’ extraction are then referred. The polymers used for the fabrication of indicators are also listed, along with the physical properties of the final materials and how these are affected by the addition of anthocyanins. Most importantly, in this review, the dependence of the colorimetric performance of the polymer-based indicators on their structure and morphology is critically scrutinized, highlighting the fact that the appropriate structural characteristics improve the sensibility and accuracy of these systems. Apart from the pH-indication, the antioxidant and antimicrobial properties of anthocyanins [45] and their incorporation in polymeric systems for the fabrication of smart materials with controlled release of their active component are evaluated. Last, but not least, the current challenges of the combination of anthocyanins with polymeric systems are discussed in order to stimulate future research for the improvement and creation of functional and competitive materials for the market.

## 2. pH Indicators: Main Application Fields

### 2.1. Food Packaging

Every year, one third of the produced food (1.3 billion tons per year) ends up wasted before being consumed, as it never leaves the production sites, gets lost or spoiled during distribution, or is discarded by the end users. At the same time, about 815 million people live with chronic hunger [55]. Moreover, food loss and waste deplete the environment of the already limited natural resources as the energy and sources for its production, transport, and packaging are wasted. Therefore, apart from the ethical aspect, food loss and waste also have a significant environmental impact, as about 8% of the total anthropogenic greenhouse gas emissions could be reduced if they were prevented [56]. To deal with this issue, recent targets to be achieved by 2030 [57] are set by the United Nations (UN) member states, by introducing the Sustainable Development Goals (SDGs). Among them, SDG12 “responsible consumption and production”, deals directly with actions for the reduction of food loss and waste, while others are indirectly connected with this initiative (e.g., SDG2 “zero hunger”) [55].

The non-appropriate storage and/or transfer conditions in the whole supply chain may easily result in food contamination and spoilage, with mainly, the oxidation and microbial growth to cause a reduction in the food shelf life and an increased risk of foodborne diseases upon consumption [58,59]. The expiration date present on packages is not always representative of the food condition. The food spoilage may occur prior to this date due to inadequate storage or the product may be still appropriate for consumption even after the expiration date. These facts bring up the necessity for real-time monitoring of aliments in order to precisely indicate their condition. Considering that most of the food waste (*ca.* 50%) is produced in the households [60], in the latest years, a vast variety of freshness indicators were developed that are able to denote the state of the food by a simple visual color change. The majority of the developed indicators are made of polymeric matrices incorporating active components, integrated in intelligent and/or smart food packaging systems. What is meant by intelligent packaging is the informative, responsive packaging, able to integrate the concept of communication with commonplace packaging, providing the consumer with information about the current situation of the packaged product [61,62]. In contrast, smart packaging refers to a corrective responsive packaging system, able not only to play the role of monitoring and communication but also able to prolong the product’s shelf life by releasing active agents [62].

A common way to track food spoilage in real-time is by monitoring the pH modifications caused by the alteration of the physicochemical conditions of the food during its shelf life. Specifically, due to inappropriate storage, transportation or packaging conditions, oxidation or microorganisms already present or introduced in the food which start growing in an uncontrolled way, cause the degradation of proteins and fats. This leads to the production of various metabolic gases such as carbon dioxide (CO_2_) or alkaline nitrogenous substances, known as total volatile basic nitrogen (TVB-N) [23,31]. Their presence alters the pH of the food itself but also of the package’s headspace, and these changes can be traced by the colorimetric indicator, conveying information about the food’s quality and making the consumer able to “read” such information on the package. Phenolic compounds such as anthocyanins, not only have an indicative pH evaluation ability, but also offer a wide array of additional properties, such as antioxidant and antimicrobial, contributing to the preservation of the food itself [63,64].

### 2.2. Wound Healing

Wound infections may negatively affect the wound healing process and need continuous monitoring to prevent the deterioration of the healing that may even lead to the necrosis of the tissue or amputation [65]. Specifically, among the stages of wound healing, the inflammation and proliferation appear to be the most critical ones. This because during these phases that, depending on the type and severity of the damage, can last from 4 to 21 days, the wound is exposed to the environment and, due to the presence of wound exudates, the area becomes an ideal environment for the growth of bacteria that can alter or decelerate the healing process [66].

Under normal conditions, the healthy skin has a pH regulation barrier function that keeps its values in a mildly acidic range (pH 4–6) [67]. However, in the presence of infections, the local pH of the wound site rises to alkaline values (pH 7–9) and in some cases can reach even the extreme value of pH 10.5 where the wound fails to heal (chronic wounds) [68,69]. Therefore, the exact knowledge of the pH of the wound leads to the direct monitoring of the healing process and permits the timely and appropriate medical care in case of infections. Nonetheless, up to date, the number of scientific works on materials able to monitor the wound healing process is still limited and they will be discussed in the next paragraphs.

## 3. Sources and Extraction of Anthocyanins

### 3.1. Sources

Anthocyanins, produced via the flavonoid pathway in the cytoplasm of the colored plant cells [70], are present in various fruits, vegetables, seeds and flowers. Such plants have different anthocyanin content and natural-occurring colors, mainly due to the environmental conditions during their growth (e.g., light, temperature, pH of soil, environmental stress) and the coexistence with other pigments, such as carotenoids and other flavonoids, and minerals [71]. The pristine color of the anthocyanins depends not only on these factors, but also, after their extraction, on the surrounding environment where they are re-introduced [8,72]. For instance, when incorporated in a polymeric matrix, anthocyanins may give to the final composite a different color from the one of the initial anthocyanin extract. Therefore, the color range upon pH changes of the anthocyanin-based polymeric composites depends on the concentration of the extract [73], the background color of the polymer matrix [72], the pH value that predominates in the starting polymeric mixture and the structural constraints that the matrix may apply to the molecules [8].

The colorimetric indicators described in the literature mostly use anthocyanins directly extracted from natural occurring sources, while in some cases, also commercial anthocyanin powder was used [74,75]. In Table 1 are reported the sources, types, and pH-depended color transitions of anthocyanins, described in the main studies of anthocyanin-based polymer composites, fabricated during the last decade for food packaging applications. So far, red cabbage is the most popular source, followed by purple sweet potato. Both of them are widely available, have a great anthocyanin content, high stability and a wide transition of colors when exposed to different pHs [76,77]. To take advantage of a wider source of anthocyanins for the development of more effective and stable colorimetric indicators, combinations between anthocyanins of more than one source [78,79], and between anthocyanins and other natural pH-sensitive pigments such as curcumin [80,81], betacyanins [82] and shikonin [83] were investigated.

The origin of anthocyanins can play a role not only in the color of the material and pH-induced hue range, but also in the sensitivity towards pH changes. It has been reported that herbal extracts with a high content of acylated anthocyanins, such as black carrot, display color changes in less than 1 min in the presence of buffer solutions of different pH values, while extracts with low amounts of acylated anthocyanins, such as black beans, have higher sensitivity, with color changes taking place within 5 s [127,144]. These outcomes suggest that acylation provides the anthocyanins with an essential stabilizing effect through intramolecular interactions, resulting in an increased color stability towards the pH changes [145].

### 3.2. Extraction

Anthocyanins are soluble in polar solvents, such as water, acetone, ethanol and methanol [91]. They are mostly extracted from their origin by solvent extractions ameliorated by stirring, shaking, or sonication, or alternatively with Soxhlet extraction [146,147], and the total amount extracted is estimated by the total monomeric anthocyanin content (TMAC) or by measuring the total anthocyanin content (TAC) as described by Halász and Csóka [85] and Merz et al. [107], respectively. The six main anthocyanidins usually present in most of the sources have different polarities affecting their solubility in various solvents and therefore their extraction process. Specifically, the polarity is higher for delphinidin and gets lower for the remaining ones with the following order: cyanidin, petunidin, pelargonidin, peonidin, and malvidin [45]. When anthocyanins are not directly connected with a sugar (anthocyanin aglycones), they are more soluble in alcohols; when they are bonded to a sugar (glycosylated anthocyanins), they are more soluble in water [45]. For this reason, in the majority of the cases, alcohol solvents or a mix of them with water were used in order to extract all of the containing anthocyanins/anthocyanidins. In fact, compared to the use of only water [76,100], alcoholic solutions have been more effective for extraction as they can readily interact with a higher amount and variety of anthocyanins [117]. Furthermore, in many of the protocols, the addition of a small amount of hydrochloric acid (HCl) to the extraction medium has been reported since it tends to shorten the extraction time due to the denaturation of the cellular membranes of the plant tissues [129,148]. Nevertheless, it is always to be taken into consideration that apart from the way of extraction, the concentration of the obtained anthocyanins also depends on the source, as noted in Table 2 [149]. However, some of the sources with a high concentration of anthocyanins are not so abundant and widely available in nature, and therefore are often not considered in the anthocyanin-based indicator studies.

## 4. Anthocyanin Polymer Composite Indicators

**Figure 3:**
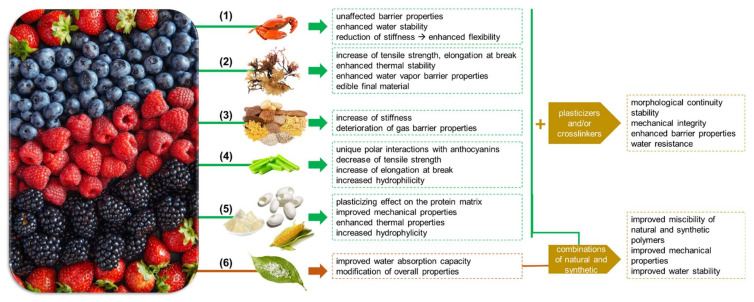
Types of anthocyanin polymer composites and their properties. Anthocyanins combined with a. natural polymers: (1) chitosan, (2) carrageenan, (3) starch, (4) cellulose, (5) proteins, or b. with synthetic polymers (6), as well as with combinations of natural with synthetic polymers and with plasticizers and/or crosslinkers. Image of berries representing anthocyanin sources reproduced from Encyclopædia Britannica, search query “anthocyanins”, https://www.britannica.com/science/anthocyanin#/media/1/27352/252532 (accessed on 22 September 2022).

An ideal indicator should present not just color changes induced by environmental stimuli, but also other features critical for the overall applicability, responsivity and stability of the colorimetric device. In fact, depending on the target application of the developed indicator, the mechanical and barrier properties, but also its fate after being used have to be evaluated. For instance, the required mechanical properties may be different if the material should constitute a free-standing composite indicator or if it should be incorporated inside an already existing packaging structure. Instead, for the design of a wound-patch indicator, a more flexible structure should be requested in order to be applied on different skin areas [66], while an indicator applied in wet environments could be a water stable and stiff material in order not to deform over time [150]. Gas barrier properties can also be a meaningful parameter that can be tuned in function of the application, since high barrier properties can be required for a fully integrated intelligent food packing system, while for the treatment of the skin, a certain range of barriers should be respected to not negatively affect the wound healing process [151]. This section aims to be a helpful guideline for the selection of polymeric matrices for the design of indicators suitable for different applications. Therefore, an overview of the currently used polymers for the production of the indicators is given below (Figure 3), and a discussion on how anthocyanins can affect the physicochemical characteristics of the final polymeric composites is reported.

### 4.1. Extraction

#### Natural Polymers

In the last years, polymers derived from natural sources are of great research interest due to the urgent necessity to switch towards more sustainable materials. Their advantageous features have been thoroughly studied and they are currently entering in the market as a promising alternative to petroleum-based plastic. Under this concept, also polymer-based colorimetric pH indicators have been designed by utilizing natural polymeric components. Most of the natural polymers ensure the production of biocompatible and biodegradable materials, two mandatory parameters for food packaging and biomedical applications, while at the same time promote sustainability and circular economy [152,153]. However, the overall physicochemical properties of the natural polymers (e.g., reduced stability in humid environments, low mechanical properties, low gas barrier properties, etc.) may compromise their use in some application fields; therefore, they should be specifically adapted through diverse combinations of materials and fabrication processes in order to meet the standards of the final commercial application.

*Single components.* Based on this strategy, natural polymers have been proposed as odorless, tasteless, colorless and sustainable carriers of anthocyanins [95] to produce all-natural indicators mainly for food packaging applications. Specifically, the use of chitosan [75,85,90,93,129,131,135,143,154], cellulose [73,106,127,133,144], carrageenan [87,108,114,155] and starch [22,94,95,96,97,101,109,126] has been reported.

One of the most widely used natural polymers as a matrix for anthocyanin-based composites is chitosan. It is firmly demonstrated that, although no significant changes in the barrier properties of the polymer have been reported, the direct interaction between the polyphenols present in the anthocyanin extracts and the hydroxyl/amino groups of chitosan through hydrogen bond interactions enhances the water stability of the composite, hindering the polymer-water interaction [75,85,90,131,135]. Furthermore, such type of interaction may cause the reduction of the overall stiffness of chitosan [135], indicating that the content and composition of the anthocyanin extracts play an important role on the modification of the materials’ mechanical properties. Enhanced water stability and flexibility of the chitosan-anthocyanin composites may be favorable for smart food packaging applications but also for an advanced component for wound management applications where chitosan is widely used also due to its antimicrobial and antifungal capacity [156,157].

On the other hand, the hydrogen bond interactions observed when anthocyanins are combined with starch [109] induce the opposite effect, leading to a slight increase in the stiffness of the polymer, and a deterioration of the gas barrier properties, due to the interactions between the water molecules and the polymer, and more specifically the water molecules diffusion that can be influenced by factors like pores, void spaces, and preferential channels through the polymer matrix [96,97]. Based on these findings, the starch-anthocyanin systems are mainly recommended as indicator labels rather than full-packaging materials. 

Carrageenan is also considered a suitable immobilization matrix for anthocyanins [155,158]. Indeed, low concentrations of anthocyanins can increase the tensile strength, elongation at break, thermal stability [87,108] and water vapor barrier properties of carrageenan-based films [114], thanks to the intermolecular interactions between the anthocyanins and the –SO3^−^ groups of the polymer [87]. Furthermore, being an edible polymer, carrageenan is a good candidate for the design of free-standing indicators to be used in direct contact with food and be consumed with it [159]. 

The use of cellulose in various types [73,106,127,144,160,161] for the development of anthocyanin-based indicators has also been widely reported. Since anthocyanins naturally occur in the tissues of fruits and vegetables, which are cellulosic environments, cellulose is an appropriate and promising matrix for them. Indeed, polar interactions between the anthocyanins and the −OH of cellulose derivatives have been noted [73,127,133,144]. affecting the intermolecular interactions of the polymer and inducing a plasticizing effect to the cellulose matrix with a decrease in the tensile strength and an increase of the elongation at break of the overall composite [73,133].

The use of protein based polymers as matrices for the anthocyanin composites has been explored, too. For instance, zein, a hydrophobic protein derived from corn [72], gelatin [99,117,146,162], derived from the hydrolyzation of collagen, and silk [147], were mixed with anthocyanin extracts to design colorimetric indicators. However, due to silk’s high cost, zein and gelatin seem to be more preferable for the development of materials that eventually can be proposed for large scale applications. Musso et al. [117] produced gelatin-based composites and observed that when the protein matrix is combined with anthocyanins extracted by alcoholic solvents, less polar anthocyanin species are also extracted together with the polar anthocyanins components, and due to the lower affinity of such species with the gelatin molecules, they interfere to the cross-linking among polypeptide chains instead of improving it, leading to less resistant, more elongated and more water soluble films. On the other hand, when anthocyanin aqueous based extracts are combined with the gelatin, an improvement of the mechanical properties of the polymer matrix was reported, due to the interactions between the polar groups of gelatin and the hydroxyl groups of the anthocyanins, favoring the proteins crosslinking [117,163]. Additionally, the hydrophilic properties of the zein-based fibers developed by Prietto et al. [72] were correlated to the anthocyanin concentration, due to their hydrophilic nature.

*Combination of different polymers.* To develop composites with more stable physicochemical characteristics in the presence of the anthocyanins but still keep the “all-natural” strategy, combinations among natural polymers were also explored. For example, chitosan with starch [141,164], pectin [74] or cellulose [165]; and κ-carrageenan [69,102] or starch [128,136] or agarose [79,166] with cellulose and/or its derivatives [69,102,118] are some of the combinations used. Furthermore, starch with sucrose and liquid inverted sugar [100] or with xanthan gum [105], and lactalbumin (whey protein) combined with gelatin [116] were also investigated. 

Nonetheless, it should be mentioned that the addition of anthocyanins in the polymer combinations, sometimes does not ameliorate the mechanical properties required for specific applications [69]. This occurs because of the inhibition of the interaction between the polymeric chains caused by the anthocyanins [100,102]. However, with the introduction of plasticizers such as glycerol [69,78,80,87,88,90,95,101,105,108,109,110,111,114,126,128,134,136,137,146] and sorbitol [97,102], and/or crosslinkers, such as sodium tripolyphosphate, (Na_5_P_3_O_10_) [164], essential oils FDue to their porosity, fibrous and foam-bas[133,167] or metallic nanoparticles [134], materials with high morphological continuity, stability, mechanical integrity [103,122,137,168], barrier properties [134], water resistance [102,168] and even better response to pH changes [133] can be formed. 

To sum up, it should be stressed that the properties of the natural polymers are significantly affected by the presence of anthocyanins. In particular, the extraction solvents, the quality, and the concentration of the anthocyanins with respect to the polymer can profoundly modify the final physicochemical properties of the natural polymer based composites. However, through a thorough exploration of the recent studies, it can be concluded that due to the great diversity of the anthocyanin sources, the extraction processes and to the variety of types of natural polymers and their combinations, it was not possible to reveal a standard guideline of the anthocyanin effects on the natural polymer matrices. Such effects should be analyzed case by case (Figure 3) and evaluated in the function of the final application. Synthetic polymers and their combinations with natural polymers. 

A way to obtain anthocyanin based composites with more consistent overall properties is to combine them with synthetic polymers that generally have better physical properties compared to the natural polymers [169]. In particular, biocompatible, non-toxic and biodegradable synthetic polymers have been recruited for the development of anthocyanin-based colorimetric indicators. To this aim, one of the most used components is polyvinyl alcohol (PVA)—alone or combined with synthetic, such as ethylene-vinyl alcohol, polycaprolactone (PCL) and poly (ethylene oxide) (PEO) [91,112], or natural e.g., chitosan or cellulose [31,107,111,113,119,121,123,138,141,170] polymers.

As also mentioned before, anthocyanins can affect significantly the intermolecular interactions of the polymers but also the polymer-polymer and polymer-water [141,171] interactions, modifying the overall properties of the composite [111,138,141]. In fact, several studies reported the positive effects of anthocyanins when used in small quantities (e.g., less than 5% wt. with respect to the polymers) [92] in polymeric composites that favorably interact with the anthocyanins, as the interactions and therefore the miscibility of the natural and synthetic polymer components are improved [78,80,110] due to the chemical interactions between the anthocyanins and the polymer molecules. As a result, the overall mechanical properties and the water stability of the final composites are improved with respect to the pristine natural polymer components (Figure 3) [140]. On the top, the overall physical properties of the resulting composites make these materials standalone options for novel packaging systems with pH-indicative properties.

### 4.2. pH-Induced Color Changes

Together with the physical properties of the anthocyanin-based polymeric composites, their ability to change effectively color upon pH alterations is of great importance. To this end, the color changes of such systems have been explored, where the anthocyanin component is either present in the fillers, e.g., agro-waste powders of various grain sizes [96,110,172], or as molecular extracts combined with the polymers [22,31,104,173,174,175].

**Figure 4:**
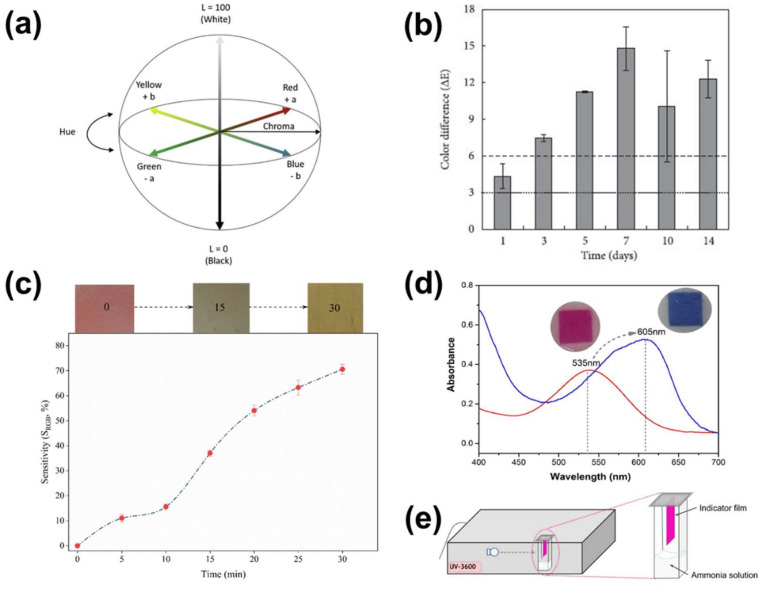
Methods of monitoring of the color change evolution. (**a**) The CIELAB color space analysis. Reproduced with permission from Ref. [177]. (**b**) The color changes of starch-based films along the monitoring of pork spoilage by using the CIELAB color space analysis. Adapted under terms of the CC-BY license [101]. (**c**) The color evolution of an anthocyanin-methylcellulose/chitosan film during its exposure to NH_3_ vapors by using the halochromic sensibility S_RGB_. The photos of the film upon exposure to the vapors for 0 min, 15 min and 30 min are also presented. Reproduced with permission from Ref. [173]. (**d**) The color change of an anthocyanin polymer composite (agarose-purple sweet potato) film monitored by UV-Vis absorption spectroscopy using (**e**) real time monitoring. Inset of (**d**): the photos of the films at the two different states. Reproduced with permission from Ref. [178].

*Methods of color change evaluation.* There are several methods to evaluate the pH-induced color changes of the colorimetric indicators. The most frequently used is the color analysis using the Euclidian distance (*dE*) derived by the CIELAB color space analysis coordinates [31,39,85,96,101,119,164], specifically designed to approximate human color perception [176]. As shown in Figure 4a, the CIELAB color space is a three-dimensional space that consists of the coordinates L, a and b. The L indicates the lightness of the sample, ranging from 0 (black) to 100 (white), while coordinates a, ranging from green (–) to red (+), and b, ranging from blue (–) to yellow (+) indicate the chroma [72]. Through the CIELAB color space analysis of the sample before and after the pH-induced color change, the color change (*dE*) can be calculated by the Equation (1):

(1) $$dE=\sqrt{\left[{\left(L_{1}-L_{2}\right)}^{2}+{\left(a_{1}-a_{2}\right)}^{2}+{\left(b_{1}-b_{2}\right)}^{2}\right]}$$

In this way, it can be quantitatively validated the color modification, showing whether a color difference can be perceived by the naked human eye [179]. Specifically, when *dE* ≥ 5, the color difference is complete and clearly perceivable, while when it is *dE* ≥ 3.5, the color difference can be noticed by inexperienced observers [179]. As an example, in Figure 4b, the dΕ color change of starch-based films along the monitoring of pork spoilage are presented [101]. The authors noticed an easily distinguishable color difference from the first day of exposure of their material to pork loin, indicating the initiation of the food spoilage process.

Alternatively, the halochromic indicator’s sensitivity (*S_RGB_*(%)) is used [173,180], which expresses how different the red (*R*), green (*G*) and blue (*B*) coordinates of the materials’ color are before and after the color modification. For the RGB analysis, all pixels within an image are split into their red, green, and blue components [176] and the averaged value of the *R*, *G*, or *B* channels is calculated by the Equation (2) [173]:

(2) $$S_{RGB}\;\left(\%\right)=\frac{\left(R_{i}-R_{f}\right)+\left(G_{i}-G_{f}\right)+\left(B_{i}-B_{f}\right)}{R_{i}+G_{i}+B_{f}}\times 100$$

where *R_i_*, *G_i_*, *B_i_* and *R_f_*, *G_f_*, *B_f_* are the initial and final values of the red, green, and blue components, respectively. Figure 4c represents the *S_RGB_* evolution of an anthocyanin-methylcellulose/chitosan composite film upon exposure to NH_3_ vapors. As noticed, the *S_RGB_* increases for prolonged exposure to the vapors, indicating the pronounced color change of the films. 

Another method for the evaluation of the changes of the optical properties of the anthocyanin based colorimetric indicators is the optical properties (e.g., absorbance) monitoring in real time using UV-Visible spectrophotometry [178] (Figure 4d,e). 

Although both RGB and UV-visible spectroscopic studies effectively show the color modification of the films, they do not represent the perception of the color changes by the human eye [31,85,176]. This is a crucial parameter that should be taken into consideration when evaluating a colorimetric indicator, since a color change is not always perceivable by an inexperienced user [181]. In order to understand whether the color changes of the colorimetric indicators are visually perceivable by the human eye, CIELAB color space analysis should be adopted as it is specifically designed to approximate human color perception [176]. Once the experimental verification that the color change is perceivable by a human observer is reached, no hardware or technological tools are needed for the assessment of the deployed materials, since the assessment can be done by naked eyes [181].

## 5. Structure-Performance Dependence

The vast majority of the up-to-date anthocyanin-based indicators are in the form of films, mainly due to their facile fabrication and utilization. Because of their structure, they can also become excellent water and oxygen barriers [93], and therefore, these so-called intelligent films offer the possibility to combine the indicating properties of anthocyanins and the adequate physical characteristics of the polymers [75,100,117] for an ideal intelligent packaging system. However, limited studies on the overall performance of such materials in terms of hydrophobicity, mechanical, and/or barrier properties exist. Therefore, in most cases the produced films are proposed solely as indicating labels [119,133,166].

Apart from the ability of a material to change color in different pH environments, an effective pH colorimetric indicator should give the color information in a specific time frame and should also be able to trace certain concentrations of volatile vapors that e.g., the food produces during spoilage, through easily perceivable color changes.

The evaluation of the color-changing ability of the developed colorimetric indicators upon immersion in buffer solutions of different pH [73,88,91,93,97,117,119,168,182,183,184] is certainly an important characterization step, but not the only one that needs to be taken into account. This is due to the fact that the direct contact of the material with the liquid does not always simulate the realistic case in which the spoilage occurs. In fact, the spoilage process not only alters the pH of the solid aliment, but also of the environment around the food. Thus, the indicator should also be able to trace this change also in a non-contact mode, through efficient interactions with the gas phase elements released in the package headspace.

*Compact film* vs. *fibrous structures.* To this end, the morphology of the indicator plays a very crucial role to their responsivity towards the elements of interest. In particular, the lack of porosity and, therefore, the low active surface area of the films [185], significantly affects their indicating ability in terms of sensitivity and responsivity, and this may lead to misleading information about the real condition to be monitored. More specifically, in most of the cases of polymer-based indicators in the form of films, the gases produced during food spoilage cannot easily penetrate their surface, leading to a slow interaction with the englobed active molecules and therefore to slow color change, thus higher concentrations of the spoilage gases are needed by the film indicators in order to sense their presence. In fact, the response time perceived as color change occurs in a timescale ranging from a few minutes to a few tens of minutes upon exposure to different pH values through the immersion in buffer solutions [78,117,127,138,147,183] or in solutions of volatile or biogenic amines such as Trimethylamine (TMA) [186], Histamine and Tyramine [187], exposure to acid and/or alkaline vapors [32,108,117,138,140,141,147], vapors produced by food [78,108,127,138,141], or upon the direct contact with food [183].

**Figure 5:**
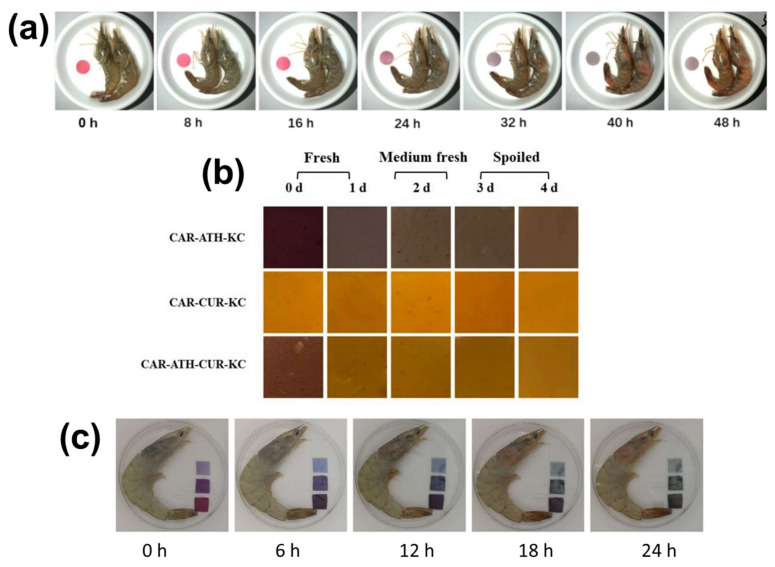
(**a**) Color modification of anthocyanin-based cellulose films during monitoring the spoilage of shrimps. Reproduced with permission from Ref. [189]. Non-obvious color changes of (**b**) anthocyanin and/or curcumin-based glucomannan/carrageenan films with for the identification of medium fresh and spoiled chicken. Reproduced with permission from Ref. [81]. (**c**) Anthocyanin-based starch/PVA films with three different concentrations of anthocyanins between the critical hours (6–18 h) of the spoilage of shrimps. Adapted with permission from Ref. [78].

In most of the studies, after the exploration of the overall properties of the developed colorimetric indicator films and their color change in contact with the buffer solutions, tests in vapor environments of wide pH ranges [117,122,129], with food simulants [96] and real food such as olive oil [104], milk [103,119,188], meat [101,106,143,166], fish [80,111,127,128,143,164] and shrimps [107,108,112,138,144] were conducted. However, the materials were not always able to warn the observer about an impending spoilage [81,189,190,191,192,193,194]. As an example, Wen et al. [189] observed the color change of anthocyanin-based cellulose films by placing them in the headspace of a container with shrimps and reported that the films change their color after 32 h of exposure. This observation was simultaneous with the color change of the shrimps from brown/grey to red, indication in itself of the quality alteration (Figure 5a). The same was noted for the films developed by Yan et al. [191] and Chen et al. [192] with shrimps and hairtail fish, respectively, for the bilayer films fabricated by Zhang et al. [193] with griskin, and for the films of Singh et al. [194] upon testing with milk. Additionally, Zhou et al. [81] explored the color change of anthocyanin/curcumin-based glucomannan/carrageenan films for fresh, medium-fresh and spoiled food, with the colors corresponding to the two latter situations being rather similar, due to the fact that the altered color hue is not distinguishable from the original one (Figure 5b). This is also observed in other similar studies [172,195,196], as in the case of Niu et al. [172], whose starch-based films’ color ranged from lighter to darker pink upon exposure to NH_3_ vapors. The material was able to indicate the spoilage of pork after 1–2 days of interaction at 4 ⁰C, something that can lead to the conclusion that although spoilage can be detected, the indicator is not able to take an intermediate color in order to warn the consumer that the food is close to spoilage and has to be eaten soon. Thus, the presence of colorimetric indicators in the packaging headspace is not always able to provide reliable, real-time information on the evolution of the spoilage process of the food.

Moreover, although the majority of the studies prove that the developed colorimetric indicators change color in the presence of spoiled food, only few correlate the color change to the production of TVB-N [78,79,88,128,137,141,167,191,197] and total mesophil counts (TMC) [126,165] during spoilage, using standard food quality evaluation in order to better understand the sensibility of the developed material. As demonstrated in such studies, the film-based colorimetric indicators were not indicating the real condition of the foodstuff due to their insufficient detection limit (i.e., the lowest concentration of a substance that an indicator can trace) [98,120,182,195,198,199]. For instance, Zhang et al. [78] used anthocyanin-loaded starch/PVA films to indicate the spoilage of shrimps, after placing them on the headspace of the packaging system for 24 h at 25 °C. According to the monitored concentration of the produced TVB-N, the shrimps were already unacceptable for consumption after 18 h of storage. However, no obvious color change of the colorimetric indicator occurred at that time (Figure 5c). Furthermore, the anthocyanin-based cellulose/chitosan films developed by Tirtashi and coworkers [165] only showed distinguishable color changes upon dipping in milk stored at 20 °C for 48 h and not earlier.

**Figure 6:**
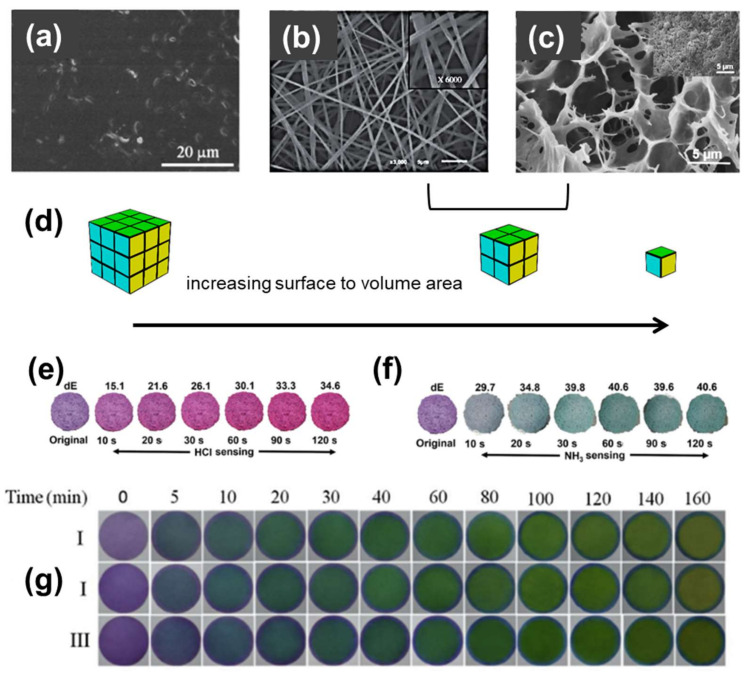
SEM images of (**a**) polyvinyl alcohol/okra mucilage polysaccharide-based film containing rose anthocyanins. Reproduced with permission from Ref. [138], (**b**) zein-based fibers containing red cabbage anthocyanin extract. Reproduced with permission from Ref. [72]. (**c**) PVA/PVP/cellulose-based foams containing red cabbage anthocyanin extract. (**d**) The increasing surface to volume area with the increase in porosity. pH-induced color evolution over time of the (**e**) foams exposed to HCl vapors, (**f**) foams exposed to NH_3_ vapors. (**c,d**) Reproduced with permission from Ref. [31]. (**g**) Films exposed to NH_3_ vapors. Reproduced with permission from Ref. [138].

Therefore, it can be concluded that film-shaped indicators generally have a slow response to the pH changes. More importantly, in the case of low concentrations of off-flavor compounds, film structures are not able to effectively detect the pH changes, meaning that their detection limit is rather high, limiting their use. All these facts can be attributed to the low active surface area of the film-indicators, which makes the permeation of the vapors though the polymer, and therefore the interaction with the responsive molecules, herein the anthocyanins, quite difficult. As a result, the response time, and therefore the time needed for the film to change color, is increased, and at the same time also the detection limit, i.e., the lowest concentration of vapors that they can trace, is negatively affected as not all the embedded responsive molecules can interact effectively with the spoilage products (Figure 6a,d).

Therefore, porous structures that can allow the penetration of the gases of the metabolic activity of microorganisms are needed for these types of application. Their advantages derive from their high surface area and mainly focus on three criteria: the real-time response of the indicator, its capacity to present color changes that are perceivable by the human eye, and its ability to respond even when small concentrations of gases are present (low detection limit). Indeed, a recent study by Kossyvaki et al. [181] showed that the more porous—thus with higher surface area—the indicator is, the lower is the detection limit and the faster and more intense the color change upon exposure to amine vapors. More specifically, a direct comparison was made between two different PCL/curcumin based fibrous structures—one with intrafibrillar pores on/in the fibers and one without them—by exposing them to vapors of dimethylamine exactly under the same conditions. CIELAB color space analysis showed that the more porous structure was able to react faster to the presence of the vapors (27.4 s compared to 103.8 s to reach the same dE value), obtaining a stronger color change (dE = 56.0 compared to 32.4). Moreover, the porous fibers presented higher sensitivity and responsivity to the vapors, with a distinct color change at very low concentrations (2.33 ppm compared to 9.26 ppm) within the first 5 s of exposure. These findings occurred because the porous fibers had *ca.* 50% higher surface area than the non-porous equivalents (8.1 m^2^g^−1^ compared to 4.3 m^2^g^−1^), thus the interaction with the amine vapors was much easier in the first case.

Hence, alternative structures have been proposed such as anthocyanin-based surface coatings [112,118,147], or fibrous mats and foams with a high surface to volume ratio (Figure 6d) and porosity [23,31,72,91,113,116,118,147,181,200,201,202], which allow faster response and higher sensitivity with respect to compact films fabricated by the same starting materials [202] (Figure 6b–d). Due to their porosity, fibrous and foam-based colorimetric indicators do not present high barrier properties, so it is difficult to be proposed as an integrated smart packaging system. Therefore, in most of the cases such materials have been proposed as pH indicating labels, while highly stable fibrous materials can also be used in integrated multilayered films envisioning novel smart packaging systems. Nonetheless, also in such cases, as well as in their use as wound dressings, materials with good mechanical properties and suppleness should be targeted [66,150].

The most popular technique to fabricate porous anthocyanin-based indicators is the electrospinning [72,91,113,116,118,201], while the incorporation of anthocyanins into foams [31] or the surface functionalization of fibrous mats and woven fabrics with anthocyanin extracts [118,147] are also studied. For the electrospun mats, in most of the cases the anthocyanins embedded in the polymer matrix increased stability and decreased susceptibility to degradation due to the protective role of the polymer, resulting in long-lasting and effective indicators [35]. Besides uniaxial electrospinning [72,91,113,118,201], fibers can also be fabricated by coaxial electrospinning [116,203], a technique that provides a core-shell structure, with the core material composed of the anthocyanins being protected from the environmental factors that cause its oxidation [116]. Porous materials can also be produced in the form of foams, by various fabrication methods [204], such as freeze-drying in the case of Zia et al. [31], with the anthocyanins’ stability being tuned according to the thermal, water absorption and other properties of the used polymers [31].

The quick and evident response in pH changes of materials with porous morphology and enhanced surface area is a crucial aspect also confirmed by recent studies [73,88,91,98,144,168,201]. Zia et al. [31] noticed an evident color change of their anthocyanin-loaded PVA/cellulose foams when exposed to HCl (dΕ =15.1) and NH_3_ vapors (dΕ = 29.7) even after only 10 s of exposure (Figure 6e,f). In contrast, a similar PVA/mucilage polysaccharide material with a compact film structure presented much slower color changes, as the first visible color alteration was observed after 5 min of exposure in volatile ammonia (Figure 6g) [138]. In the same work, composites with a higher concentration of anthocyanin presented a more intense color, but also a bigger response time due to the color hysteresis phenomena. The latter occurs because of the higher amount of anthocyanin molecules, which preserve for longer time the original color of the composite even though some other anthocyanin molecules already undergo structural transformation in the presence of the different pH environment.

Although the detection limit studies of the anthocyanin-based indicators are scarcely reported, recent studies explore the lowest concentration of gases that a certain material can trace. For instance, Sun et al. [98] demonstrated that their PLA/anthocyanin-based fibers can trace down to 37 ppm of ammonia, a value that according to the authors’ findings permits the material to effectively sense the spoilage of fish in real time. This is a very important aspect of the evaluation of a colorimetric indicator, since its responsive capacity has to be in accordance with the quality limits of e.g., fish and meat for human consumption [205].

**Figure 7:**
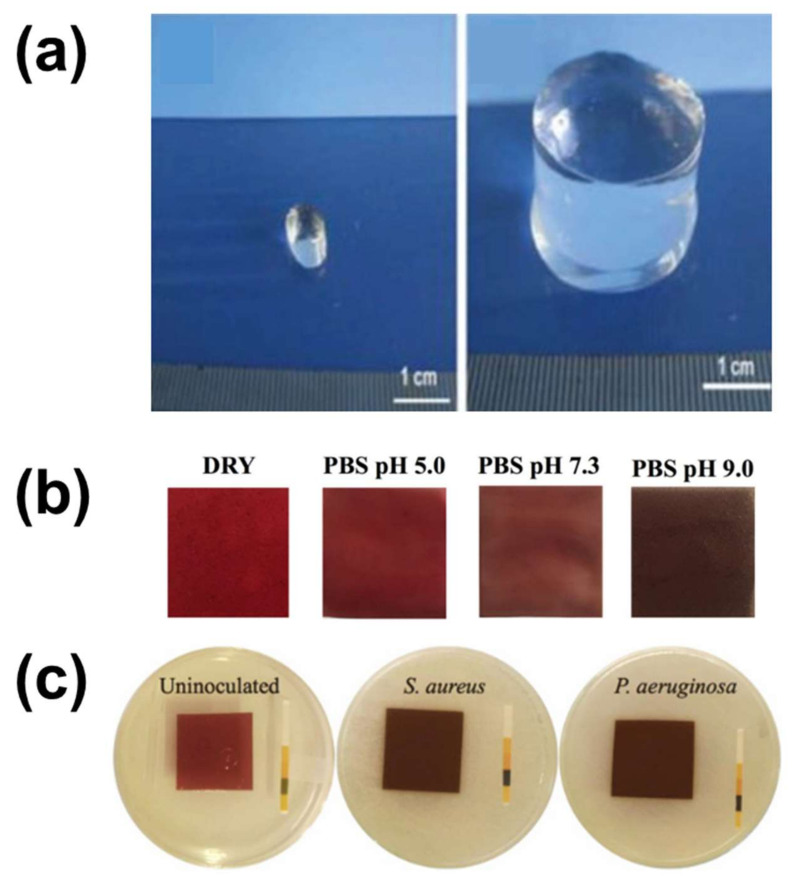
(**a**) The swelling of a nanocomposite hydrogel that is able to maintain its structural integrity [207]. Adapted under terms of the CC-BY license. Color changes of carrageenan/gum-based hydrogel containing cranberry extract (**b**) upon immersion into buffer solution of different pH, relevant to skin wound pH values; (**c**) upon exposure to bacteria. (**b**,**c**) Adapted with permission from Ref. [69].

*Other Structures.* Apart from the fibrous mats and the foams, hydrogels are also gaining growing interest in the field of porous colorimetric indicators. Hydrogels are a category of three-dimensional polymer networks consisting of macromolecular chains linked through chemical bonds, and their most important characteristic is that they can absorb and retain large quantities of water or aqueous fluids [206] maintaining their structural integrity, as shown in Figure 7a [207]. Hydrogel based materials have been developed for various applications including food packaging [208], biomedical [209] and water technologies [207,210,211,212], and as carriers of bioactive compounds. The incorporation of anthocyanins [125,213,214] and other dyes [215] in hydrogels has been recently studied, thus the use of the hydrogel structure for the development of colorimetric indicators appears auspicious [69].

Anthocyanin-based hydrogel colorimetric indicators can be effectively used is the biomedical field in order to indicate the status of the wound during healing. In fact, hydrogels can interact efficiently with the humid environment of the wound and therefore permit to the loaded anthocyanins to indicate the pH of such an environment through their color change. Recently, Zepon and coworkers [69] developed an anthocyanin-based carrageenan/gum hydrogel for the colorimetric monitoring and prevention of bacterial infections in the wound bed. After being immersed into buffer solutions of different pH values in order to demonstrate its color change capacity (Figure 7b), the hydrogel got exposed to *Staphylococcus aureus*, one of the most commonly occurring pathogens in the natural and hospital environments, and to *Pseudomonas aeruginosa,* a foodborne bacterial strain that can also be met in the aforementioned environments. When in contact with these microorganisms, the color of the anthocyanin-loaded hydrogel changed, indicating the modification of the pH of the wound environment [201] to a value representative of an infection, as seen in Figure 7c.

Based on this interesting work, the combination of the superabsorbent properties of the hydrogels, with their porous structure, and the indicating properties of the anthocyanins could lead to innovative, smart materials. These advanced systems could be able to monitor the growth of the bacterial population in various environments and, at the same time, they could control the humidity of the system by absorbing the excess and releasing the anthocyanins in a controlled way, giving them the opportunity to act not only as indicators but also as active compounds, as will be discussed in the next section.

## 6. Functional and Active Anthocyanin-Based Polymeric Systems

As described until now, the anthocyanin-based polymeric systems are mainly utilized as colorimetric indicators. However, recently, researchers are also focusing on the possibility of using these systems as advanced active materials, by taking advantage of the other functional properties of anthocyanins, such as their antioxidant and antimicrobial activity.

In fact, several studies have shown the excellent ability of the anthocyanins to give strong antioxidant activity to the polymeric composite systems in which they are included. This property is linked to their structural and intrinsic electronic features and distribution, with the phenolic hydroxyl (−OH) group to be the main protagonist of this mechanism, and is mostly quantified by performing DPPH• and ABTS• radical scavenging assays [104,135,163,216]. To explain the free radical scavenging ability of anthocyanins, two mechanisms have been proposed: the single electron transfer (ET) (3) and the hydrogen atom transfer (HAT) (4). In both cases, the obtained phenolic radical is stabilized due to the π-electrons in the aromatic ring of the anthocyanins [217,218].

ArOH + R^●^ → ArOH^●+^ + R− (3)

ArOH + R^●^ → ArO^●^ + RH (4)

Anthocyanins also have antimicrobial activity due to their capacity to disrupt the cell wall and membrane, to interact with the mechanisms of protein synthesis or increase protein degradation, and to bind the DNA [219]. Therefore, the inclusion of anthocyanins in the polymeric systems can give them the additional functionality to act against bacterial growth. Various studies based on biobased films (e.g., κ-carrageenan [114], chitosan [171]) loaded with anthocyanins have demonstrated such activity by being tested with various types of bacteria such as *Escherichia coli*, *Salmonella*, *Staphylococcus aureus, Listeria monocytogenes,* and *Pseudomonas aeruginosa*. However, even if the anthocyanins can be effectively used as antibacterial agents for foodborne bacteria, it is worth noting that they do not affect the viability of *Lactobacillus rhamnosus* [220], an intestinal bacterium type involved in the digestion process, indicating the possibility to be safely used in food-based applications. To further improve their antibacterial action, anthocyanins are combined with other antibacterial components such as silver nanoparticles, or polymers (e.g., chitosan) [134]. Moreover, combinations of the anthocyanins with weak organic and phenolic acids [221,222,223] can cause multiple bactericidal mechanisms such as alterations of the cell membrane integrity and binding to genomic DNA to inhibit cellular functions [223]. Therefore, the antibacterial performance of anthocyanins can be improved through a potential synergy among components that may derive from the same extract [220,224].

To take advantage of their antioxidant and antibacterial properties as well as their pH indicating ability, anthocyanins should be able to indicate the modification of the food environment and to diffuse from the polymeric system to the external environment only when this change occurs, in order to directly provide the food with efficient antimicrobial and antioxidant actions. In fact, the activation and release of anthocyanins should occur only when required; for instance, during microbial proliferation and/or significant changes in the food condition (variation of pH or production of ethanol).

So far, only a few studies investigated the release property of the colorimetric indicator composites. The release should be evaluated in environments that simulate the potential alterations of food in the packaging system, such as excessive humidity, alcoholic environments or environments with different acidity. Most of the reported studies are based on anthocyanin polymer composite films and, depended on the polymer matrix type, they have shown the capability to retain the anthocyanins for up to 4–12 h after immersion in different solvents with a slight dependence of the release profile to the solvent type [108,225,226]. This outcome suggests how the different solubility and diffusion properties of the anthocyanins in the different environments, when englobed in a polymer matrix of a specific nature, can affect the overall performance of the responsive material and define targeted use for specific applications.

**Figure 8:**
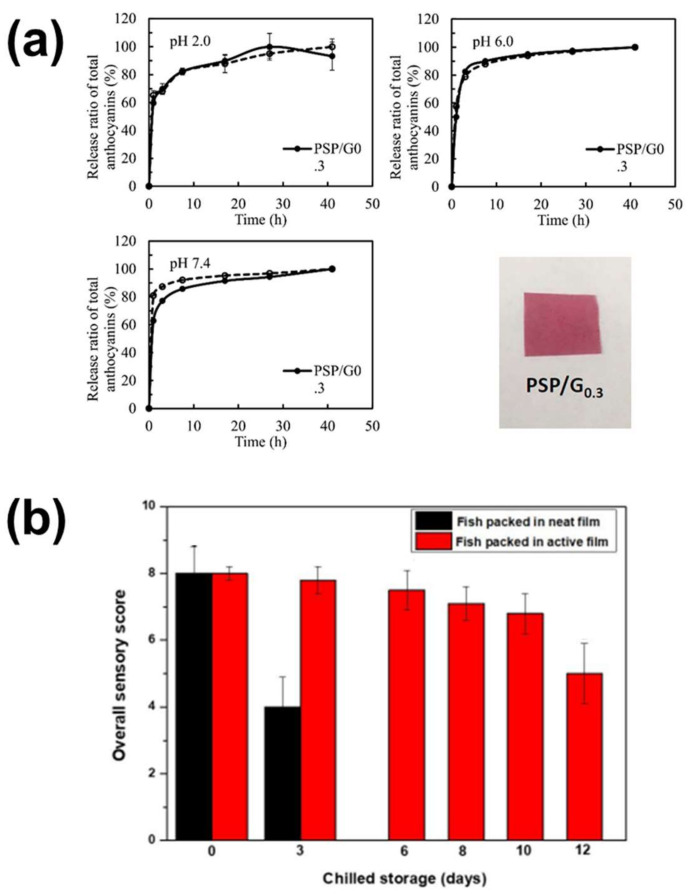
(**a**) The gellan gum/purple sweet potato-based films (PSP/G_0.3_) and their 40-h-long release profiles in different pH conditions (2, 6, and 7.4). Interactions between the gellam gum and the phenolic compounds resulted in a slower release profile for pH 2. Adapted with permission from Ref. [130]. (**b**) The color, odor and texture sensory scores of packaged fish without and with the application of active cellulose/PVA based films with rose petal anthocyanins, which manage to conserve it for 12 days. Adapted with permission from Ref. [227].

In fact, the nature of the polymer matrix can significantly affect the diffusion and responsive release properties of the anthocyanins. Specifically, the choice of the polymers—e.g., natural, biodegradable, edible –, their intrinsic features—e.g., hydrophilic or hydrophobic–, the type of their interactions with the anthocyanins—e.g., chemical or physical—and their structural properties—e.g., porous or compact films—can affect the controlled release and introduce novel perspectives for the developed colorimetric indicators. For example, Wei et al. [130] highlighted the exciting possibility of having an edible pH-indicator that could also act as a potential delivery system for the anthocyanins inside the human body. Such edible pH-indicator film based on gellan gum/purple sweet potato can be used directly in contact with the food and subsequently consumed together with the food itself (Figure 8a).

However, only in the pharmaceutical field, it is well-known how the composite’s structure, such as films, gels, foams, fibers and particles, can strongly affect the final release profile and the diffusion of a “small molecule” from the polymeric matrices [228,229,230,231,232,233,234]. For food packaging related studies, although it has already been evaluated the release profile from films and particles as vehicles for anthocyanins, no works reporting a comparison between the different polymeric structures, their release profile, and their capacity to prevent food degradation are present [124,137,163,225].

As previously reported, the combination of antibacterial, antioxidant and pH indication ability of the anthocyanin-loaded samples can lead to a fully integrated smart system able to indicate and preserve the status of the aliment or the wound. A step towards this concept has been performed by Kanatt [227], who developed a cellulose-PVA based film loaded with rose petal anthocyanins to monitor the released TVB-N from fish samples during the spoilage process. The developed material was not only able to indicate the spoilage, but also to control the bacterial growth and proliferation, decelerating the process which, according to the sensory scores (based on color, odor and texture) measured, could be effectively preserved for 12 days, instead of its pristine shelf life of 3 days when stored without the presence of the anthocyanin films (Figure 8b).

To sum up, the advanced properties reported in this paragraph can be suitable for the design of functional anthocyanin polymeric-based materials capable not only of acting as passive monitoring tools but also to interact directly with the food. Such advanced materials can indicate and delay the spoiling process of food and subsequently, depended on the type of the polymer matrix, be consumed together with the aliment or biodegrade, solving thus the problem of potential waste production. Moreover, these features can be exploited in other areas such as biomedicine, going towards the direction of multifunctional and multitasking biomaterials.

## 7. Current Challenges and Future Research

*Anthocyanin stability.* The main challenge for the use of anthocyanins in the field of pH indicators is their relatively low stability. It has been found that monomeric anthocyanins are extremely unstable and can be easily degraded to colorless or brown-colored compounds, losing their pH indicating ability [132,235]. Structure also plays an important role in the anthocyanins’ stability, with pelargonidin, cyanidin and delphinidin being less stable than peonidin, petunidin and malvidin due to the “blocked” reactivity of the ortho-hydroxyl groups by the methylation (−OCH_3_) that the last three present [236], as it can be noticed in Figure 1. Furthermore, anthocyanins stability is affected by environmental factors, including temperature, light, and by the presence of other phenolic compounds, enzymes, metal ions, sugars, ascorbic acid, and oxygen [145]. Specifically, their stability decreases with the increase in pH, temperature and sugar content [160], and studies recommend their use mainly at low temperatures, such as the ones of chilled products [237].

A way to deal with this situation could be the extraction from sources that mainly contain acylated anthocyanins, such as sweet potato and purple carrot, since acylated anthocyanins show improved stability due to the steric hindrance of phenolic acyl groups which reduces the susceptibility to water attack and prevents the subsequent formation of colorless hemiketal and chalcone forms [238]. Acylation can be also chemically induced to the anthocyanins by solid-phase grafting methods using maleic anhydride or eventually other protective substances [238]. As a result, such types of anthocyanins are more resistant to color fading with increased pH than their unacylated analogs, such as red grape extract [239], while the final colorimetric indicator would present higher stability and long-lasting performance. Another strategy is the combination of anthocyanins with non-polar polymers or polymers with high oxygen barrier properties, which can protect the interaction of the active molecules with the water, and therefore the long-term stability of the colorimetric indicator can be improved [91,112].

**Figure 9:**
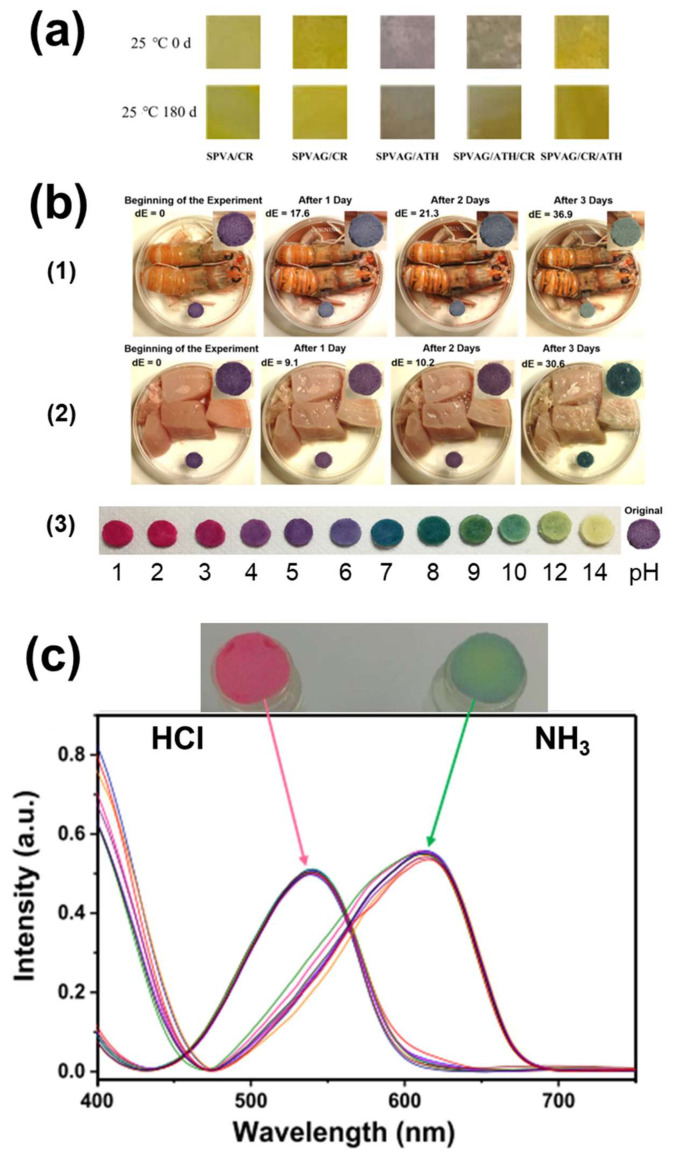
(**a**) Stability studies of PVA-starch based films with or without glycerol (SPVAG and SPVA, respectively) and with different combinations of curcumin (CR) and anthocyanins (ATH) (SPVA/CR, SPVAG/CR, SPVA/ATH, SPVAG/ATHCR, SPVAG/CR/ATH curcumin:anthocyanin ratio of 1:0, 1:0, 0:1, 2:8 and 8:2, respectively): images of the films the first and the 180th day of storage at 25 °C. Adapted with permission from Ref. [80]. (**b**) Monitoring of spoilage of (1) prawns and (2) chicken using the red cabbage anthocyanin-loaded foams, (3) and their wide pH-induced color change. Adapted with permission from Ref. [31], (**c**) Optical images and UV–vis reflectance absorption spectra of anthocyanin dyed silk fabrics exposed to 8 cycles of HCl and NH_3_ vapors. Adapted with permission from Ref. [147].

It has also been reported that the stability of the anthocyanin pigment can be enhanced by combining it with another pH-sensitive pigment such as curcumin [80]. As demonstrated in Figure 9a, the higher the curcumin concentration in PVA-starch-glycerol films, the higher the stability of the anthocyanins loaded in them. This can be attributed to the fact that molecules of bigger size, such as curcumin, enhance the stability of the anthocyanins due to effect of copigmentation, which protects them from taking the form of chalcones that are more susceptible to color fading [240]. Copigmentation can occur also in presence of polysaccharides, such as alginate and chondroitin sulfate. In such case, the interactions between the polymer and the anthocyanins create a supramolecular polymer that protects the flavylium cation from a nucleophilic attack (e.g., from water), but also form more intense colors with respect to anthocyanins alone [241,242].

*Indication accuracy: Real-time monitoring and detection limits.* In most of the already published scientific works, it has been noted that systematic studies of the detection limits and real-time monitoring of pH changes are missing. In fact, as reported in Section 5, some authors are focused either on the color behavior of the anthocyanin extract and not of the final polymer composite indicator or on the colorimetric assay of the material when immersed in buffer solutions of different pH values. However, these results are often not representative of the behavior of the material when in contact with the vapors released from the foodstuff during spoilage, impeding thus their accuracy and subsequently their large-scale commercialization.

To overcome these problems, more precise experiments should be conducted, on the interactions of the colorimetric indicator with the vapors released during food spoilage or upon the direct contact with the source of spoilage. Shukla et al. [79] suggests the testing of the materials directly with the metabolites produced by specific microorganisms found in each food instead of testing with the general quality indicators, such as TVB-N. This could be a more precise method, targeted to specific foods because in each food different spoilage processes may occur producing different byproducts—the so-called off-flavor compounds. In such case, more precise information on the quality of specific food (i.e., fish and meat, milk, fruits and vegetables) would be extracted, offering the possibility to create indicators with enhanced performance for defined foodstuff.

In addition to the studies on specified metabolic products representative of specific food products, the utilization of porous materials would result in highly sensitive colorimetric indicators, as stressed in the Section 5. In fact, the volatility of the amines calls for an immediate tracing of such vapors in the packaging headspace at concentrations relevant to the spoilage, and the few studies on porous colorimetric indicators have demonstrated their ability of doing so. More relevant studies concerning the interaction of porous materials with vapors of amine-based metabolic products are performed on curcumin-loaded composites, rather than anthocyanin-based materials [23,181]. In fact, ethyl-cellulose poly(ethylene oxide) and polyvinyl pyrrolidone nanofibers loaded with curcumin, were tested in six different volatile amine analytes (ammonia, TMA, DMA, TEA, Piperidine and Hydrazine) to study the color change capability of curcumin and the limit of detection and quantitation [23]. Results showed that the material was able to present a color change depending on the type of the present amine, whereas minimal color changes at low concentrations of TMA (10, 20 and 40 μg/mL) and NH_3_ (3 and 6 μg/mL) vapors were observed. However, the rate of the color change and the testing with real food are not reported.

In another recent work, Zia et al. [31] performed kinetic studies in order to define the sensibility of their porous indicators towards pH changes induced by HCl and NH_3_ vapors (Figure 9b). Results were analyzed with CIELAB color space analysis and compared with spectroscopic analyses, while the porous material was also tested with real food (prawns and chicken, Figure 9b). Color changes were visible after just 10 s of exposure and covered a wide range of pH values (1–14) (Figure 9b). However, neither the detection limit nor the interaction with the various microorganism metabolites/amines were investigated.

The combination of the two lastly described works could be a potential guideline for a more precise and detailed investigation of the kinetics of the color changes and of the sensitivity towards specific substances. The interaction time and the correlation of the color change behavior with specific concentrations of substances representative of the food spoilage or wound condition are the key points towards the development of accurate indicators. Moreover, the response of the material to very low concentrations of vapors [243,244] and generally, the exploration of the detection limit is another important criterion that should always be included in such works. By better understanding the indicating potential of the anthocyanin-based polymeric materials, their introduction to the market for direct utilization in smart packaging systems would become possible.

*Reversibility.* The reversibility of the colorimetric indicators, namely the ability of the material to recover its initial color after its use, is another aspect that needs to be considered, depending on the final application. For instance, in the case of food packaging, a colorimetric material can be offered either as an indicator kit that can be used independently from the packaging system, or as an already included component of the package itself. In the first case, the reusability of the material is of major importance, since in this way the end-user can always use the same indicator every time is needed to control the status of a stored food, whereas in the second case, an indicator with irreversible color changes could be more reliable, in order to prevent the provision of false negative results in case of packaging leakages.

The color reversibility of some of the already mentioned indicators has been studied upon the application of a specific treatment [31,108,117,120,147,245]. For instance, Tang and coworkers [147] tested the reversibility and reusability of the developed colorimetric pH-sensing silk fabrics by exposing them to 8 cycles of HCl vapor alternated with NH_3_ vapor, proving an immediate color change between bright red and yellow—green (Figure 9c). Although this indicates the possibility of the reutilization of the colorimetric indicator for multiples times, precise stability testing in the presence of the biogenic gas metabolites, but also upon specific storing conditions with the food, have to be performed, in order to verify the stability of the anthocyanins for long enough to be reused multiple times [118].

*Future research.* With the research on polymeric colorimetric indicators containing anthocyanins, there are still a lot of paths to explore. One of the targets of future research could be the use of anthocyanin sources studied but not incorporated in indicators yet. For instance, anthocyanins extracted from red autumn leaves [246], produced by microorganisms [247], or blue anthocyanins bio-synthetized from genetically engineered tobacco cell suspension culture [248], may arise as alternatives to the anthocyanins from food sources that are currently used, in order to avoid using food sources for applications other than nutrition.

Another goal should also be the use of the whole anthocyanins source as it generally contains various natural polymers, such as cellulose and starch that can be also used for the fabrication of a totally natural indicator, as starting material for the fabrication of indicators, either as it is or by extracting all the necessary components from the same source. For example, Ishak and coworkers developed a polymeric film using starch extracted from purple sweet potatoes along with anthocyanins of the same source [216]. A sustainable target could be the utilization of the part of the plant that is considered as “waste”, such as the peels, roots and leaves. The concept of using the waste of an edible source instead of the source itself has been gaining popularity during the last years, mainly due to the ethical issue of how food sources should be used [249]. Such waste type sometimes is even 50% of the total weight of the source and can include bioactive compounds ideal for the fabrication of innovative sustainable materials [94]. This could also pave the way for a more sustainable and innovative waste management. Indeed, grape [102,103], mulberry [250] and blueberry [93,94,95,97] residues from wine and juice production, respectively, have been successfully used as source for the fabrication of all-natural colorimetric indicators.

For all the materials developed, a step that should not be omitted is their biocompatibility or cytocompatibility in order to confirm the suitability of the fabricated materials for use in food and/or skin applications. For instance, Singh et al. [194] performed the cytocompatibility tests of their film samples on human keratinocyte skin cells and erythrocytes. In this way, the potential toxicity of the materials can be evaluated and consequently the safety for the consumers can be ensured.

Future research should also include the design of more sophisticated polymer composite systems, able not only to respond to the alteration of the environment by changing their color, but also to provoke the release of the anthocyanins at specific conditions in a controlled way, thus prolonging the antioxidant and antibacterial actions given by them, and thus improving the overall performance of the smart indicator, as discussed in Section 6. 

An additional future perspective could be the expansion of the fields of application of the anthocyanin-based pH indicators. Currently, and as described herein, most of the related studies deal with food spoilage and packaging. However, the (micro) environments in which pH alterations may occur are much more, and so the fields of application, with the examples of the drinking water [251] and protective clothing and textiles [252,253]. The use of natural molecules such as anthocyanins to develop materials for these fields could be of great importance since limitations of chemical pH indicators, such as the toxicity, would not be an obstacle for their commercialization. In addition to this and as already mentioned, the pH indicative property of anthocyanins could be combined with their health-promoting activities to make materials that could actively contribute to the overall performance.

Finally, yet importantly, the incorporation of anthocyanin-based polymeric materials in microfluidic systems [67] and electronics [176] could result in devices that can function not only as indicators but also as precise sensors, giving punctual quantitative information. In this way, communication with the end-users would be easier, and these materials could become more attractive for the market.

## 8. Conclusions

In conclusion, anthocyanins appear as promising molecules for the development of multifunctional pH colorimetric indicators. Their appropriate inclusion in polymeric composites can improve their stability and preserve their pH-induced hue range. Furthermore, together with the right combination of polymer matrices and depending on the targeted application, the final system can exhibit improved overall performance such as mechanical and barrier properties. On the top, owing to the intrinsic properties of the anthocyanins, together with the color change response to different pH environments, additional functionalities can be evaluated such as the antioxidant and antimicrobial activity. As highlighted in this review, the structure of the polymeric matrices plays a crucial role in the pH-indicating performance of the incorporated anthocyanins and thus in the material’s sensibility. More targeted and thorough research on the kinetics of pH-induced color changes and on the materials’ detection limit of microbial metabolites’ concentrations should be conducted, while the role of antioxidant and antimicrobial activity in the food preservation should be better investigated. Such findings can pave the way for the next generation of smart materials for a more sustainable future.

## Figures and Tables

**Table 1:** Sources of anthocyanins, their types, and color behavior from representative studies focused on food packaging applications [51,84,85,86,87,88,89,90].

Source	Anthocyanin Type	pH-Dependent Color Transition		
		Acid	Neutral	Base
Açai [91]	cyanidin, delphinidin, malvidin, pelargonidin, peonidin, petunidin	Red	Pink/purple	Purple/grey
apple (*Malus pumila* P. Mill.) [92]	Cyanidin	not reported	not reported	not reported
blackberry (*Rubus fruticosus* agg.) [93]	Cyanidin	not reported	not reported	not reported
blueberry (*Vaccinium corymbosum* L.) [93,94,95,96,97,98]	Cyanidin, delphinidin, malvidin, peonidin, petunidin	Pink	Purple	Brownish
cranberry (*Vaccinium macrocarpon*) [69]	Cyanidin, delphinidin, malvidin, peonidin	Pink	Purple	Brownish
black chokeberry (*Aronia melanocarpa*) [85]	cyanidin, pelargonidin	Red	Purple	Brown
dragon fruit [86,99]	not reported	Light pink	Pink	Yellow
grapes (*Vitis vinifera*) [8,100,101,102,103,104]	petunidin, malvidin	Pink	Light purple	Green—yellow
jabuticaba flour (*Plinia cauliflora*) [105]	not reported	Pink	Purple	Brown
jambolan or jamun fruit (*Syzgium cumini*) [106,107]	delphinidin, petunidin, malvidin	Red/violet	Violet blue	Green—yellow
*Lucium ruthenicum* Murr. [108,109]	cyanidin, malvidin, peonidin, petunidin, pelargonidin	Pink	Purple	Blue—yellow
Mulberry [87,110,111,112]	cyanidin, malvidin, pelargonidin	Red	Purple	Grey
Pomegranate [113,114,115]	cyanidin, delphinine	Light orange	Dark brown	Light brown
sour cherry (*Prunus cerasus* L.) [116]	Cyanidin	not reported	not reported	not reported
red cabbage (*Brassica oleraceae*) [31,73,78,79,117,118,119,120,121,122,123]	cyanidin, pelargonidin	Red	Blue	Orange—yellow
black carrot (*Daucus carota* L.) [124,125,126,127]	cyanidin, delphinine	Red	Pink	Orange—yellow
purple and black eggplant (*Solanum melongena* L.) [90]	cyanidin, delphinine	Pink/red	Blue/purple	Beige/yellow
purple sweet potato [78,99,128,129,130,131,132,133]	cyanidin, peonidin	Pink	Pink	Green
purple corn (*Zea mays* L.) [134]	Cyanidin, pelargonidin, peonidin	not reported	not reported	not reported
purple and black rice (*Oryza sativa* L.) [88,135,136,137]	cyanidin, peonidin	Pink	Purple	Bluish black
Rose [79,138]	Cyanidin, peonidin [139]	Pink	Red	Yellow
Roselle [86,140,141]	Cyanidin, delphinidin [142]	Dark pink	Pink	Yellow
*Bauhinia blakeana Dunn.* Flower [143]	not reported	Red	Pink	Green
*Echium amoenum* flower [144]	not reported	Bright red	Indian red	Green
Butterfly pea flower [86]	not reported	Red	Blue	Blue

**Table 2:** Total anthocyanin content of various anthocyanin sources [149].

Source	Total Anthocyanin Content (mg/kg)
Fruits	
**Apple (peel)**	100–2160
**Bilberry**	4600
**Blackberry**	820–1800
**Blueberry**	825–5300
**Cherry**	3500–4500
**Chokeberry**	5060–10,000
**Cranberry**	460–2000
**Currant (black)**	1300–4000
**Currant (red)**	119–186
**Elderberry**	2000–15,600
**Grape (red)**	300–7500
**Grape (blue)**	80–3880
**Plum**	19–250
**Raspberry (red)**	100–600
**Raspberry (black)**	80–3880
**Strawberry**	127–360
**Vegetables**	
**Cabbage (red)**	250
**Eggplant**	7500
**Onion (red)**	up to 250
**Radish (red)**	110–600
**Rhubarb**	up to 2000

## Data Availability

Not applicable.
